# Need for recovery in relation to effort from work and health in four occupations

**DOI:** 10.1007/s00420-019-01476-7

**Published:** 2019-10-16

**Authors:** Kerstin Wentz, Kristina Gyllensten, Judith K. Sluiter, Mats Hagberg

**Affiliations:** 1 Occupational and Environmental Medicine, University of Gothenburg and Sahlgrenska University Hospital, Box 414, 40530 Gothenburg, Sweden; 2 Coronel Institute of Occupational Health, Amsterdam UMC, Academic Medical Center, PO Box 22700, 1100 DE Amsterdam, The Netherlands

**Keywords:** Recovery, Work-related fatigue, Psychosocial work characteristics, Occupations, Stress, Rumination

## Abstract

**Objective:**

To examine three levels of need for recovery (NFR) after work in relation to effort from work demands, demand compensatory strategies, effort-moderating or -reversing resources, and health including health behaviors. A further purpose was to examine occupational characteristics determining NFR.

**Methods:**

5000 engineers, carpenters, nurses, and home care nurses were invited to participate. NFR k-means clusters were calculated from 1289 participants. The effect from three levels of NFR regarding demands, compensatory strategies, resources at work, health, and health behaviors was examined using analysis of variance (ANOVA) and post hoc analysis. Prevalence ratios (PRs) of suboptimal health for three levels of NFR were calculated using Poisson regression. Linear stepwise multiple regression predictors explaining NFR were examined also occupation wise.

**Results:**

NFR centroids at 5.8/33, 13.1/33, and 21.0/33 points were identified. ANOVA showed corresponding effects from NFR levels on work demands and compensatory strategies. The inversed proportion concerned levels of resources at work. Only the low NFR cluster negated regular health effects. The other two cluster groups also repeatedly worked while ill and presented PRs concerning health effects from 1.9 to 3.9 when compared to the low NFR group. Making good quality work, recovery opportunities, and thinking of work when off work were the most important predictors of NFR among 1289 participants with also occupation-wise interpretable profiles.

**Conclusions:**

Three levels of NFR meant corresponding levels of work demands, work-demand compensatory strategies, and unfavorable health behaviors. An inversed proportion of resources related to the same levels of NFR. Low NFR meant no regular health effects which could guide limit values regarding salutary NFR. Important predictors of NFR were resources making a good quality work, recovery opportunities, and reversely effort from rumination when off work. Occupation-wise predictors could guide interventions.

## Introduction

The relationship between self-reported unfavorable working conditions and severe health consequences such as cardiovascular disease (e.g., Kivimäki and Kawachi 2015) has been repeatedly established. This relationship also involves potential precursors, or biological risk factors, for cardiovascular disease, such as diabetes or adverse lifestyles (e.g., Nyberg et al. 2013). At the same time, the broad concept of unfavorable working conditions poses a challenge, because it embraces a substantial variety in occupations, which makes the work-related causative agents in morbidity and mortality largely unknown (Van Amelsvoort et al. 2003). In addition to the variety of working conditions in the different occupations, a single employee is likewise exposed to a variety of individual working conditions. This myriad of conditions is one of the difficulties that faces both employees and researchers in evaluating exposure to stressors at work. An important approach was, therefore, the suggestion by Van Amelsvoort et al. (2003) to capture and record exposure by shifting focus from the exposure to the severity of the stress experience instead. In so doing, exposure to stress may be translated into an experience in terms of the time frame of recovery from the exposure. This time frame could, at an intermediate level, be translated into the mental load reaction that is present at the end of the working day, termed need for recovery (NFR). The NFR load reaction was pictured by Van Veldhoven (2008, p. 3) as a “collection of symptoms, temporary feelings of overload, irritability, social withdrawal, lack of energy for new effort, and reduced performance”. That the NFR measure meaningfully represents the time frame of recovery was subsequently confirmed by Schuring et al. (2004), who mapped the effect from scheduling of free time between work shifts in the transport sector.

The effort-recovery model represents the load process of work (Meijman and Mulder 1998) and how a psychobiological response reversibility or recovery in time will reinstate baseline values. This effort-reversal process takes place both within and between the work shifts and relates to an array of available opportunities to recover. Work breaks, holidays, and beginning and ending times of the workday all represent recovery opportunities (Van Veldhoven 2008). For the next day at work to start without residual symptoms, the effort-reversal process of the worker has to be successfully completed on a daily basis (Demerouti et al. 2009). At the same time, De Lange et al. (2009) have documented an insufficiently completed process of recuperation in terms of high work demands and increasing fatigue over time. These researchers also identified long-term consequences in terms of a failing mechanism of recuperation. Moreover, Van Veldhoven (2008) described how long-term fatigue-related syndromes, for example, burnout or exhaustion, mean experiencing both a high NFR and an inability to recuperate. In line with this, the fact of two distinguishable states of fatigue—on one hand, exhaustion (Maslach et al. 1996), and on the other hand, NFR—has been confirmed (Siltaloppi et al. 2009).

The fact of job demands creating effort may be represented by workload, such as needing to work extra hard to manage the work demands, physical demands, emotional demands, and by work–home interference, such as difficulty managing domestic obligations because of scheduling (e.g. Van den Broeck et al. 2008). To these acknowledged causes of effort resulting from job demands, for example, Aronsson et al. (2013) add work demand-related suboptimal individual coping strategies, termed compensating strategies. These strategies are intertwined with completing the tasks and concern *how* this is done. They comprise, for example, *working more intensively to finish different work tasks.* These strategies seem to belong to a class of suboptimal and self-consuming coping measures in terms of work style (Feuerstein et al. 2004) and overcommitment (Siegrist 2012). Logically, these causes of effort need also to be included in the amount of effort that is the subject of the reversal process.

Van Veldhoven (2008), relying on Meijman (1989), described how different job characteristics such as situational characteristics affect how the work demands create load effects. Therefore, the total amount of cumulative effort that appears as NFR could in part be explained by the effort moderation or reversal. In this process, for example, job control plays a significant role (Van Veldhoven 2008). As well, different job resources may reduce physiological and psychological costs of the job demands by facilitating achievement of work goals and stimulating personal development (Schaufeli and Bakker 2004). The effort expenditure at work could also be said to be moderated or lightened, because the process of doing the work is in line with satisfaction of basic human needs such as feeling autonomous, or experiencing one’s own competence or a taste of community of relatedness at work (Van den Broeck et al. 2008). van Hooff and Geurts (2015) even showed how motivation was spurred from need satisfaction which saved on self-controlling mental effort which resulted in less fatigue at the end of the working day.

**Fig. 1:**
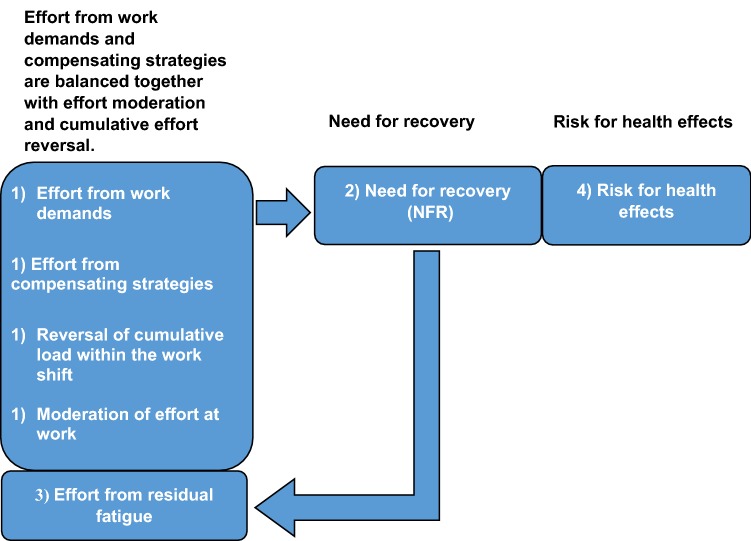
A hypothesized temporal process is that (1) the effort from work demands together with (1) effort from work-demand compensating strategies is potentially balanced together with (1) resources that are either or both effort-moderating and (1) effort-reversing in the work place. The result from effort and effort moderation and reversal at work is accompanied by (2) short-term load reactions in terms of need for recovery NFR. (3) Fatigue that is not reversed between the work shifts means effort from residual fatigue, which adds to the weight of effort from work and from work-demand compensating strategies to NFR. NFR is in turn accompanied by (4) risk for health effects

A heightened level of NFR is associated with concurrent suboptimal health (e.g., Van Veldhoven and Sluiter 2009). Both Sluiter et al. (2003) and Van Amelsvoort et al. (2003) argue in favor of recording short-term load reaction of an increased level of NFR, as these recordings may play an important role in preventing suboptimal health. Also, both concurrent suboptimal health and prevention are reasons to define an unhealthy level of NFR that is distinguished from a harmless or salutary NFR. In turn, the perspective of the model in Fig. 1 is of a temporal process, where NFR concerns recovery and holds an intermediate position between effort from work and risk for health effects. The intermediate position of NFR concerns load reactions over 3–4 weeks that may signal failing recovery process. A heightened NFR may mean additional fatigue, accompanied by additional effort at work from residual fatigue. This vicious circle of ongoing increase in the short-term load reaction means in itself suboptimal health and is also an early signal of the risk for more severe effects on health. The weight of effort from work is studied from the perspective of work demands, work-demand compensating strategies, and effort from residual fatigue. The weight from effort is parallel balanced together with the weights of the resources with potentials of moderating effort or gradually reverse accumulation of effort.

The NFR scale (Veldhoven and Meijman 1994) records work-related fatigue symptoms translated into the level of NFR after work. The scale captures both the experience of fatigue at the end of the working day and reduced ability to continue working at the same capacity (Van Veldhoven 2008). In a principal component analysis by Jansen et al. (2002), the NFR experience was found to be distinguishable from psychological distress in terms of minor psychiatric illness. Confirmatory factor analysis by Siltaloppi et al. (2009) also showed that the NFR was distinguishable from the dimension exhaustion in the Maslach Burnout Inventory (Maslach et al. 1996). Instead, a heightened NFR measure has predictive properties, such as a doubled risk of long sick leave 2 years later (de Croon et al. 2003), cardiovascular diseases (Van Amelsvoort et al. 2003), and psychosomatic complaints (De Croon et al. 2004; Sluiter et al. 2003).

Concerning a recovery process that may counterbalance demands from work, the recovery opportunities’ (RO) instrument measures job control in terms of opportunities for recovery during work hours and in the interface between work and home (Van Veldhoven and Sluiter 2009). A clear negative association between opportunities for recovery and NFR has been documented (Van Veldhoven and Sluiter 2009; Wentz et al. 2018a, b).

### Work-related fatigue and cluster analysis

Aronsson et al. (2013) used a cross-sectional design and studied the diurnal cycle of feeling rested versus feeling fatigued (in the morning, during and after the working day, after the weekend, etc.) in human service work in relation to demands and resources at work, individual compensating strategies to manage work demands, and health problems. Experimental trials with k-means cluster analysis had earlier repeatedly shown that two s.c. extreme cluster groups with one group in-between presented consequent level distinctive profiles regarding all diurnal measurement points (e.g., Aronsson et al. 2003). Logically, this means that each diurnal measurement alone could cluster wise represent or “signal” the other diurnal values for (un)completed recovery. Aronsson et al. (2013) used this 3 means cluster design where each participant was assigned to designated “recovered”, “not recovered”, or “in-between” clusters. Thereafter, these cluster groups were examined from the perspective of the whole situation of grouped individual participants. Therefore, reported working conditions, work-demand compensating strategies, and effects on health including health behaviors were linked together and associated with the grouped individuals. From this latter analysis a pattern emerged, where the “not recovered” cluster group presented “the whole chain of risk factors” from high job demands, insufficient resources to do the work including insufficient support from management. Moreover, the chain of risk factors involved a heightened level of work-demand compensatory strategies that is, a heightening intensity of work and significantly more health problems, at the same time as they were not more absent from work but instead to a greater extent worked while ill. The risk for health effects of the “not recovered” group was also comparatively multiplied. In addition, the “in-between group” also showed a tendency for poorer health. This latter finding may create an opportunity for setting a limit value concerning sufficient recovery from work.

Aronsson et al. (2013) concluded that patterns that surrounded the cluster groups meant good reasons to proceed with a longitudinal design together with an expanded scope of professions. The present study has answered the latter call, while replicating and complementing the Aronsson et al. (2013)’s study by including occupational groups both inside and outside human service work. Also, the registrations of a diurnal cycle of recovery were replaced with registration of NFR (e.g., Van Amelsvoort et al. 2003) with a signaling role (Sluiter et al. 2003).

Exposure to stress from a variety of working conditions could be captured by the measurement NFR (Van Amelsvoort et al. 2003). In Fig. 1, these exposures equal effort stemming from work demands, behavioral (demand compensatory) strategies, residual fatigue together with effort-moderating, and effort-reversing resources. The net outcome of effort is accompanied by NFR. The hypothesized process of Fig. 1 needs to be investigated by first cluster analysis (Aronsson et al. 2013) where the individual employees are grouped based on their NFR measurement and second the cluster wise corresponding levels of effort and amelioration together with levels of health effects.

### Purpose and research questions

The main objective of the present study was to examine three levels of perceived NFR after work in relation to the potentially balancing weights of effort from work demands including compensatory strategies and the effort-moderating or gradual effort-reversing resources doing the work. Furthermore, the objective concerned functional health effects and health behaviors and in a variety of jobs. Need for recovery entails feelings of tension, fatigue, and cognitive difficulties at the end of the working day. A symptom and signaling role was hypothesized for NFR between working conditions and suboptimal health (e.g., sleeplessness and depression) concerning a heightened level of NFR after work. A further aim was to examine the impact of diverse occupational characteristics on NFR.

Specific research questions were:

1. How are NFR cluster groups related to work demands and resources doing the work?
2. How are NFR cluster groups related to work-demand compensatory strategies?
3. How are NFR cluster groups related to functional health effects?
4. How are NFR cluster groups related to health-related behaviors?
5. What is the impact of occupationally characteristic on NFR after work?

## Methods

### Design

The present baseline assessment is part of a repeated measurement longitudinal epidemiological research design on how to facilitate recovery from work in three different age groups and four different occupations. The design includes also measurements of physical load, physical exertion, and pain. The examination of physical and mental need for recovery together and over time will gradually take place and be presented elsewhere.

### Participants

The selection of respondents was based on age and differential exposure to physical load, mental load, or physical and mental load. A further alignment was to include professions with high versus low rates of occupational injuries, where engineers represent a clear low rate of reported occupational injury (Statistics Sweden). The age groups were: 18–35, 36–45, and 46–70 years. The professional groups were engineers and architects with a 5-year university degree, carpenters working at building sites, nurses working in hospitals, and home care nurses working in the homes of care recipients. Random sampling was done based on the Swedish Standard Classification of Occupations (SSYK), using the above criteria and including individuals 19–70 years of age. Sampling was done using proximity sampling in the Västra Götaland Region or within a specified municipality in Västra Götaland Region.

### Procedure

In a first step, Statistics Sweden sent letters of invitation to 1250 representatives in each of the four occupational groups. The invited individuals were requested to mark “other” if their current occupational group was neither engineer nor carpenter, nurse, or assistant nurse working in home care. The letter also included a numbered consent form, the survey, and a return envelope. Interested individuals sent the consent form including their name, postal address, and social security number and the completed questionnaire back to the research group. One reminder, based on a temporary, numbered list of addressees at Statistics Sweden, was sent to those who had not responded within 2 weeks. This concluded the first phase of the longitudinal study. A total of 1292 individuals had responded (25.8% response rate). The response rate was 18%, 12.5%, 43.4%, and 8.6% for engineers, carpenters, hospital nurses, and home care nurses, respectively.

### Instruments and variables

The total survey consisted of 115 questions.

### Background

The background variables were sex, age, marital status, children, children currently living at home, type of employment, contract, and working hours (full time/part time). Concerning the number of children living at home, responses were divided into three categories: 1 = none, 2 = one or two, and 3 = three or more.

### Work effort, effort-moderating resources, and cumulative effort-reversing resources together with need for recovery, functional health effects, and health behaviors

**Fig. 2:**
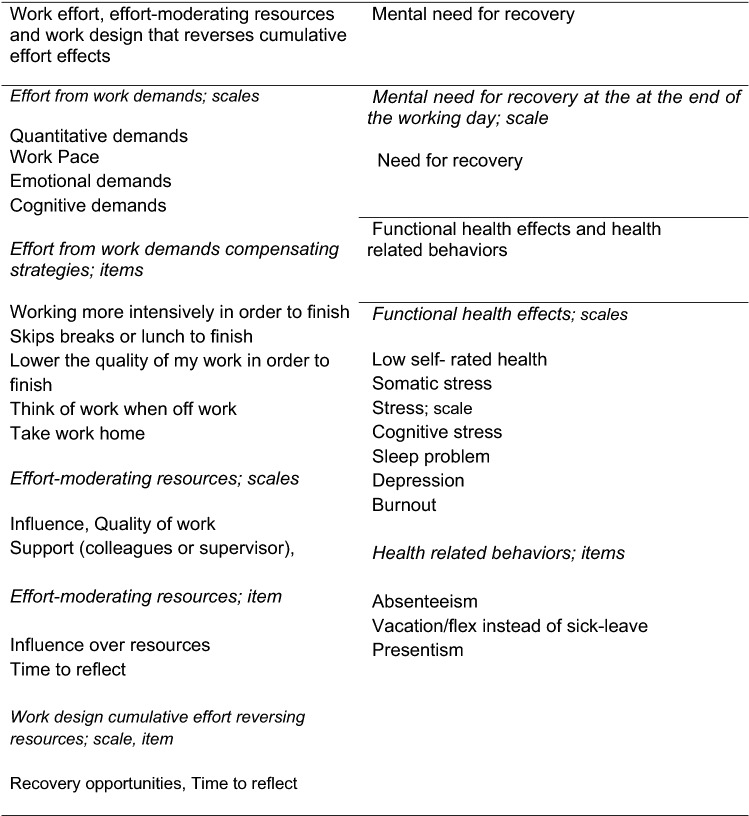
Overview of scales or single items mirroring the effort-recovery process including health effects and health behaviors

Figure 2 shows an overview of measurements described in detail below.

### Effort from work: work demands and compensatory strategies

The Swedish version of the Copenhagen Psychosocial Questionnaire (COPSOQ) adapted by Berthelsen et al. (2014) was used. The Swedish version includes the work-demand scales Quantitative demands (four items, e.g., “Do you fall behind in your work?”), Work pace (three items, e.g. “Is it necessary to keep working at a high pace?”), Emotional demands (three items, e.g. “Is your work emotionally demanding?”), The COPSOQ Cognitive demands scale (four items, e.g. “Does your work require you to remember a lot?”) was adapted by the research group (see section on health scales). The items of two scales, Work pace and Emotional demands, have five response options ranging from “To a very small degree” to “To a very high degree”. The other COPSOQ scales have five response options ranging from “Never/almost never” to “Always”. The response options for all items of the six scales were translated into 0, 25, 50, 75, and 100 points, respectively. Each scale has a total score ranging from 0 to 100 points based on the item average. Higher scores indicate a larger number of demands and resources, respectively. For details regarding these items, see Berthelsen et al. (2014).

Five single items with the same response options as for the resources at work items examine *Compensatory strategies* for handling work demands (Aronsson et al. 2013). They are: “When there is a lot to do, I work more intensively in order to finish what needs to be done,” “I skip breaks or lunch to finish what needs doing,” “I lower the quality of my work in order to finish what needs to be done,” “I take work home with me and complete it in my spare time,” and “I think of my work even when I am off work.” Higher scores indicate a more frequent use of compensatory strategies.

### Resources that moderate effort

Two work moderating resource scales were collected from Swedish version of the Copenhagen Psychosocial Questionnaire (COPSOQ) (see paragraph on demands); Social support from colleagues (three items, e.g. “How often do you get help and support from your colleagues?”), and Social support from the supervisor (three items, e.g. “How often do you get help and support from your nearest superior?”). Aronsson et al. (2013), when examining resources at work, used single items. One of these specifically concerned *Influence over resources* when doing work: “Are you and your work group able to influence how many resources for carrying out your work you will have?” Four items reflect either the moderating resource of an opportunity to deliver good *Quality of work*: “Do you have adequate resources to perform your work in a way that is satisfactory to you?” “Are you satisfied with the quality of the work you do*?*” or the opposite, “Do you ever feel inadequate because you are unable to give the help or support that you would like to give?” together with “Do you have so much to do that you don’t get around to doing as good a good job as you would like?” The single items of Quality of work compose a scale termed Quality of work. A good internal consistency was found for the scale (Cronbach’s alpha = 0.795) among 1280.

### Work design cumulative effort-reversing resources

Recovery opportunities’ (RO) scale records a load effect reversing effect. That is the degree of personal freedom regarding work shifts in terms of, for example, breaks and scheduling of shifts. In parallel to the NFR scale (see below), the RO scale has been translated from Dutch (Van Veldhoven and Sluiter 2009) into Swedish and tested in Swedish samples (Wentz et al. 2018a, b). The scale comprises nine items, for example, “Can you decide yourself when you take a break?” and “Are your working hours and free days arranged well?” The items have four response options ranging from 0 = “Never” to 3 = “Always”. Higher scores indicate greater opportunities for recovery. Good internal consistency was found for the Swedish version of RO scale (Cronbach’s alpha = 0.803) among 142 participants (Wentz et al. 2018a, b).

### Need for Recovery, functional health effects, and health-related behaviors

The 11-item NFR scale records cumulative load effects centered around at the end of the working day by asking participants to respond to items such as, “By the end of the working day I feel really worn out,” “I am able to relax only at the second day off,” and “I cannot really show any interest in other people when I have just come home myself.” The scale (Veldhoven and Meijman 1994) was translated from Dutch in accordance with the guidelines for cross-cultural adaptation of self-report measures on health, proposed by Beaton et al. (2000). In the Swedish version, the original dichotomous scale was converted into a 4-point scale ranging from 0 = “Never” to 3 = “Always”. This change means that the scale average could be expected to go down (Van Veldhoven 2008). The total score ranges from 0 to 33, where higher scores indicate a higher NFR after working time. A good internal consistency was found among 146 participants (Cronbach’s alpha = 0.901) (Wentz et al. 2018a, b).

The functional health effects were recorded using the Health and Well-being scales from the Swedish version of the COPSOQ by Berthelsen et al. (2014) or by the research group using the cross-cultural adaptation process (Beaton et al., 2000). The scales adapted by Berthelsen et al. (2014) record Cognitive and emotional expressions of *Stress*, respectively, by items such as “How often have you felt stressed?”, bodily expressions of *Problems sleeping* by asking, “How often have you slept badly and restlessly?”, and *Burnout* by using items like “How often have you felt worn out?” The scales adapted by the research group record *Depression*, for example, by asking, “How often have you lacked self-confidence?” *Cognitive stress* by asking, “How often have you had difficulty remembering”, and *Somatic stress* using items such as “How often have you had heart palpitations?”. The five response options range from 0 = “Never/almost never” to 100 = “All of the time”. Higher scores indicate worse health. The single item on *Self*-*rated health*, asks “In general, would you say your health is ____” gave five response options ranging from 0 = “Bad” to 100 = “Excellent”, where higher scores represent better health.

Health-related behaviors were measured by the Godin instrument (Godin and Shephard 1985), which records exercise habits, categorized into exerting, moderate, and light exercise, by frequency of exercising per week. Another instrument we used was the Saltin-Grimby instrument (Rödjer et al. 2012), which records the frequency, during an average week, that regular physical activity was performed intensely enough to elevate heart rate and start sweating, where 1 = “Very rarely”, 2 = “Sometimes”, and 3 = “Often”.

Health-related behaviors were also recorded in terms of absenteeism and presenteeism. Absenteeism was recorded as the number of occasions of sickness absence from work during the last 12 months, where five response options range from 1 = “Never” to 5 = “More than 10 times”. In addition, absenteeism was recorded by means of one item concerning absence from work due to sickness without reporting as sick and instead using vacation time/flexi-leave. This item had five response options, from 0 = “On no occasion” or 0 = never sick” to 3 = “More than five times”. A third measure of health-related behaviors concerned the number of times, during the past 12 months, the respondent had been present at work despite a need for sick leave, where 1 = “Never” and 5 = “More than five times”.

### Statistical analysis

Background descriptive variables of four occupational groups and the other group were calculated. The mean NFR score of the current sample of 1289 participants was calculated, and thereafter, k-means cluster analysis was carried out using (SPSS version 18, SPSS Inc., Chicago, IL, USA). The participants were assigned to one of the three clusters where the difference between the individual score and the cluster center was the smallest. The cluster 3 means analysis designated the after-work load reaction as a high, a low, or an in-between level of NFR. Regarding each cluster group, the means of NFR and of the successive scales or single items were transformed into descriptive profiles (Nunnally and Bernstein 1994) that is in the dimensions of work demands, resources doing the work, compensatory strategies, and functional impact on health, respectively. The profile-level descriptors were the response options for each scale or item.

The effect of the “low”, “in-between”, and “high NFR” clusters on background variables, work demands and resources doing the work, compensatory strategies, functional impact on health, absenteeism, presenteeism, and exercise habits was calculated using analysis of variance (ANOVA) and Tukey Honestly Significant Difference post hoc analysis. Thereafter, the risk for effects on health in terms of health and well-being scales were dichotomized in terms of frequency of complaints (Aronsson et al. 2013). The COPSOQ scales were categorized into 1 = 0–25 points (“None of the time” and “A little of the time”) and 2 = 26–100 points (“Some of the time”, “Quite a bit of the time”, and “All of the time”). Concerning the COPSOC item *Self*-*rated health*, the responses “Poor”, “Fair”, and “Good” were rated 1, while “Very good” and “Excellent” were rated 2. To establish the effect of each of the three NFR clusters on health problems, the relative prevalence (RP) of suboptimal health for each of the cluster groups was calculated through a modified Poisson regression (Zou 2004). The group with a low NFR became the reference group, with the prevalence values transformed to 1. The prevalence of suboptimal health in the other groups was presented as prevalence ratios (PRs) based on transformation of the low NFR value 1. Concerning the different occupational characteristics, the impact of work demands and resources doing the work and compensating strategies on NFR was examined. The method of use was linear stepwise multiple regression. In a second analytical step, age was added to the equation as a predictor.

### Ethical approval

The study was approved by the Regional Ethical Review Board at the University of Gothenburg as part of a five-measurement longitudinal research project on recovery in different age and professional groups from the perspective of health (Dref 050-15). Informed consent forms were sent back to the research group together with the completed first measurement questionnaire.

## Results

### Background variables

The four professions and the group labeled “other” were examined regarding background variables. As can be seen in Table 1, the proportion of men (43%) was smaller than that of women (57%). Among carpenters, nurses, and home care nurses, most participants were 46–70 years old. Among the engineers, the largest age group was the 36- to 45-year-old group. In all occupational groups, permanent employment was most prevalent. The human service occupations (nurses and home care nurses) differed from the other two groups by having a greater proportion of part-time workers, with one-third working part time as compared with 5% in the male-dominated professions (Table 1). The reason for not working full time, given by about one-third of both hospital nurses and home care nurses, was that they found work too strenuous (Table 1).

**Table 1:** Background variables of four occupational groups

	Engineers	Carpenters	Nurses	Home care nurses	Other	Total
Number in each occupational group	230	156	543	115	238	1282
Gender [*n* (%)]						
Male	177 (77)	154 (99)	82 (17)	17 (15)	122 (51)	549 (43)
Female	53 (33)	1 (1)	451 (83)	98 (85)	116 (49)	715 (57)
Marital status [*n* (%)]						
Married/cohabiting	201 (87)	133 (85)	426 (78)	66 (57)	181 (76)	1007 (79)
Living alone	29 (13)	22 (15)	117 (12)	47 (53)	56 (24)	271 (21)
Age [*n* (%)]						
18–35 years	43 (19)	26 (17)	48 (9)	32 (28)	54 (23)	203 (16)
36–45 years	106 (46)	36 (23)	142 (26)	22 (19)	67 (28)	373 (29)
46–70 years	80 (35)	93 (60)	353 (65)	61 (53)	117 (49)	704 (55)
Children [*n* (%)]						
1–2	139 (60)	88 (56)	341 (63)	47 (41)	122 (51)	737 (57)
3 or more	48 (21)	41 (26)	138 (25)	31 (27)	49 (21)	307 (24)
Children living at home						
1–2	126 (55)	60 (38)	241 (44)	23 (20)	91 (38)	541 (42)
≥ 3	35 (15)	15 (10)	43 (8)	11 (10)	23 (10)	127 (10)
Form of employment contract						
Permanent	226 (99)	147 (94)	524 (97)	104 (90)	210 (88)	1211 (94)
Temporary	2 (1)	6 (4)	16 (3)	10 (9)	24 (10)	58 (5)
Retired	1	1 (1)				621 (1)
Working time						
Full time	215 (93)	150 (96)	358 (66)	80 (70)	201 (84)	1004 (78)
Part time	14 (6)	4 (4)	182 (34)	35 (30)	33 (14)	268 (20)

Numbers in parentheses indicate the percentage of valid answers in each occupational category

This reason for working part time was almost exclusively seen in human service work (Table 1).

### Need for recovery, k-means clusters, and background data including professions

The mean NFR score for the total sample of 1289 participants was 11.6 points (standard deviation, SD 6.0). The cluster analysis centroids were identified at 5.83, 13.08, and 20.96 points for the “low NFR”, “in-between NFR”, and “high NFR” groups, respectively. The cluster groups experienced the NFR load reaction “less than sometimes”, “sometimes” “and” “often”, respectively. Altogether, the recovered group (“low NFR”) comprised 40.2%, the in-between group 41.6%, and the not recovered group, 18.1%. The low, in-between, and high NFR groups had an age mean of 49, 47, and 45 years, respectively. Regarding children, 56%, 58%, and 70%, respectively, of the low, in-between, and high NFR groups had children living at home (Table 1). Concerning full-time work, the corresponding proportions were 82%, 78%, and 72%.

The mean NFR score for architects/engineers was 9.5 (SD 5.5), for carpenters 10.5 (SD 5.3), for hospital nurses 12.2 (SD 6.0), and for home care nurses 14.2 (SD 6.7). The relative frequencies of engineers, carpenters, nurses, and home care nurses with a low NFR were 49%, 48%, 37%, and 26%. The corresponding figures concerning a high NFR were a 13%, 15%, 19%, and 30%, respectively (Table 2).

**Table 2:** Need for recovery (NFR) in terms of the clusters “low NFR”, “in-between NFR”, and “high NFR”, by profession

	Engineers	Carpenters	Hospital nurses	Home care nursed	Total including Other
*N*	230	156	543	115	1265
Low NFR (%)	112 (49)	75 (48)	200 (37)	28 (26)	509 (40)
In-between NFR (%)	84 (37)	58 (37)	232 (43)	45 (39)	526 (42)
High NFR (%)	29 (13)	23 (15)	103 (19)	35 (30)	230 (18)

## How are NFR cluster groups related to work demands and resources doing the work?

The between-group ANOVA showed a significant effect of the NFR group with regard to all conditions of work. The high NFR group reported the highest demands and the least available effort-moderating or effort-reversing resources at work. This meant, e.g., reporting the least satisfaction with having enough resources to “do a good job” (*Quality of work scale*) (Table 3). The low NFR group presented the opposite pattern. The NFR groups differed significantly from each other, with the exception of the *Support from colleagues* scale, concerning the high NFR group versus the in-between NFR group. Among the effort modifying resource scales concerning *Influence*, *Support from supervisor*, and *Support from colleagues* and the work-demand scale *Cognitive demands*, a small increment in mean values was seen from the low NFR to the in-between to the high NFR group. The effort-reversing RO scale showed a statistically significant difference between the NFR groups, with values aggregating in the middle of the scale.

**Table 3:** The effect of three levels of need for recovery (NFR) after work on work demands and resources that moderate effort at work and resources that reverse cumulative load effects

Scale or single item	Low NFR group, mean	SD	In- between NFR group, mean	SD	High NFR group, mean	SD	*df* between groups, and within groups	*F* value	*P* value
Work demands									
Quantitative demands (1–100)	**30.6**	**17.5**	41.6	19.5	**50.2**	**21**	2, 1254	94.77	0.000^a^
Work pace (1–100)	**47.3**	**19.1**	57.4	18.5	**67.2**	**18.3**	2, 1257	96.541	0.000^a^
Emotional demands (1–100)	**40.8**	**26.4**	52.7	24.8	**64.9**	**25.5**	2, 1256	74.291	0.000^a^
Cognitive demands (1–100)	**68.3**	**17.5**	72	16	**75.0**	**16.2**	2, 1257	14.703	0.000^a^
Resources that moderate effort									
Influence (1–100)	**50**	**21**	42	19.6	**35**	**20.4**	2, 1253	45.692	0.000^a^
Single item Influence over resources (0–4)	**1.7**	**1.2**	1.4	1.1	**1**	**1.1**	2, 1251	32.775	0.000^a^
Support from colleagues (1–100)	**65.9**	**19.2**	59.1	18.5	**56.9**	**18.7**	2, 1251	24.660	0.000^b^
Support from supervisor (1–100)	**64.3**	**23.1**	57.4	21.6	**51.9**	**23.3**	2, 1248	26.195	0.000^a^*
Quality of work (1–16)	**12.3**	**2.3**	10.3	2.5	**8.2**	**2.9**	2, 1253	2013.507	0.000^a^
Single item time for reflection/discussion (0–4)	**2.5**	**1.1**	2.0	**1.1**	**1.6**	1.1	2, 1259	62.88	0.000^a^
Work design cumulative effort-reversing resource									
Recovery opportunities (RO) (0–27)	**17.3**	**5.2**	14.4	5.1	**12.1**	**4.7**	2, 1241	90.141	0.000^a^

The table presents means and standard deviation (SD) on the scales and in single items for each NFR group, with ranges in brackets. The measurements from the two extreme cluster groups are presented in bold numbers

^a^The effect of NFR was significantly different between all three groups, with *p* ≥ 0.000

^a^*The effect of NFR was significantly different between the groups, with *p* ≥ 0.000, except for the difference between the high NFR group and the in-between NFR group where the level of significance reached *p* ≥ 0.05

^b^The effect of NFR was significantly different between the high NFR group and the low NFR group and between the low NFR group and the in-between NFR group, with *p* ≥ 0.000. The difference between the high NFR group and the in-between NFR group was n.s

## How are NFR cluster groups related to work-demand compensatory strategies?

Some compensatory strategies were clearly more frequently used than others. Concerning the whole sample (*N* = 1285–1287), the means and (standard deviations) were in size as follows: working more intensively 2.8 (1.0), thinking of work when off work 1.9 (1.2), skipping breaks 1.7 (1.3), lowering the quality of work 0.9 (1.0), and taking work home 0.9 (1.7). The between-group ANOVA showed a significant effect of NFR group on strategies to increase efforts concerning work when handling work demands (Table 4). Regarding all items, all three NFR groups had a range of response options between “Fairly rarely” and “Fairly often”. Regarding the item “When there is much to do I work more intensively,” the joint main response was “Fairly often”, with the high NFR group approaching the level of “Very often”/“Always”. The item on thinking of work when being off work showed a range of mean responses between “Sometimes” and “Fairly often”, with also the largest difference between the low and high NFR groups. The high NFR group gave a mean response of “Fairly often”.

**Table 4:** The effect of three levels of need for recovery (NFR) on *Compensatory strategies* when handling work demands

Single items range (0–4)	Low NFR group, mean	SD	In- between NFR group, mean	SD	High NFR group, mean	SD	*df* between groups, and within groups	*F* value	*P* value
Compensating strategies									
When there is much to do I work more intensively	**2.5**	**1.1**	2.9	1.0	**3.3**	**0.77**	2, 1259	56.296	0.000^a^
I skip breaks to finish what needs doing	**1.2**	**1.1**	1.8	1.2	**2.1**	**1.2**	2, 1257	60.868	0.000^a^
I lower the quality of work to finish	**0.5**	**0.8**	0.9	0.9	**1.4**	**1.2**	2, 1258	68.532	0.000^a^
I take work home	**0.69**	**1.0**	1.0	1.2	**1.04**	**1.3**	2, 1258	12.483	0.000^b^
I think of work when off work	**1.4**	**1.1**	2.0	1.1	**2.6**	**1.1**	2, 1257	84.635	0.000^a^

The table presents mean scores and standard deviation (SD) in single items, with ranges in brackets. The measurements from the two extreme cluster groups are presented in bold numbers

^a^The effect of NFR was significantly different between all three groups, with *p* ≥ 0.000

^b^The effect of NFR was significantly different between the high NFR group and the low NFR group, and between the low NFR group and the in-between NFR group, with *p* ≥ 0.000. The difference between the high NFR group and the in-between NFR group was not significant (n.s.)

## How are NFR cluster groups related to functional health effects?

The between-group ANOVA showed a significant effect of NFR group on health effects and health behaviors (see Table 5). Profile-wise, the low NFR group showed a self-rated health mean that could be worded as “Very good”, while the mean self-rated health of the high NFR group was “Good”. Concerning the health scales on effects on health, the group mean of the low NFR group indicated that there were no regular effects on health, with a range from “Never” without reaching “A little of the time”. At the same time, the high NFR group tended towards “Some of the time”.

**Table 5:** The effect of three levels of need for recovery (NFR) on scores for health scales; self-rated health, insomnia, burnout, stress, depression, somatic stress and cognitive stress

Single item (score range)	Low NFR group, mean	SD	In- between NFR group, mean	SD	High NFR group, mean	SD	*df* between groups, and within groups	*F* value	*P* value
Self-rated health (1–5)	**3.9**	**0.8**	3.4	0.8	**3.1**	**0.9**	2, 1256	83.833	0.000^a^
Insomnia (0–100)	**17.9**	**15.8**	29.7	19.5	**38.1**	**21.2**	2, 1257	109.342	0.000^a^
Burnout (0–100)	**20.5**	**12.7**	38.3	16.0	**54.8**	**18.8**	2, 1256	426.718	0.000^a^
Stress (0–100)	**19.9**	**14.3**	39.0	17.3	**51.9**	**18.9**	2, 1258	343.615	0.000^a^
Depression (0–100)	**17.4**	**14.8**	34.1	17.4	**48.1**	**17.9**	2, 1258	300.814	0.000^a^
Somatic stress (0–100)	**15.0**	**13.2**	27.8	16.4	**48.1**	**17.9**	2, 1260	186.524	0.000^a^
Cognitive stress (0–100)	**18.2**	**15.5**	34.2	17.5	**47.4**	**19.1**	2, 1261	258.865	0.000^a^

Mean scores and standard deviation (SD) are given for all scales and single items. The measurements from the two extreme cluster groups are presented in bold numbers

^a^The effect of NFR was significantly different between all three groups, with *p* ≥ 0.000

In examining the relationships between level of NFR and prevalence of health effects, the variables age and occupational group were controlled for. The low NFR group was the reference group for calculations of PRs transformed to 1 (Table 6). The PRs for the in-between and high NFR groups ranged from 1.9 to 3.2 and from 2.4 to 3.9, respectively. The order of magnitude for the relationships between the NFR level and the PRs was substantially similar between the two groups. The lowest PRs were seen for self-rated health (1.9 and 2.4) and sleep problems (2.4 and 3.3). The largest effect from level of NFR in both groups concerned burnout. Divergent from the order of magnitude of these two groups was the proportionally greater magnitude of stress in the in-between group compared to the high NFR group (Table 7).

**Table 6:** Prevalence ratios (PRs) with 95% confidence intervals (CIs) for low self-rated health, somatic stress, stress, cognitive stress, sleep problems, depression, and burnout as dependent variables and level of need for recovery (NFR) after work as predictor variable

Health^a^	Low NFR group	In-between group	CI	High NFR group	CI
	PR	PR		PR	
Low self- rated health	1	1.9	1.6–2.2	2.4	2.0–2.8
Somatic stress	1	2.6	2.1–3.3	3.8	3.1–4.8
Stress	1	2.9	2.4–3.4	3.5	3.0–4.1
Cognitive stress	1	2.6	2.1–3.3	3.8	3.1–4.8
Sleep problems	1	2.4	2.0–2.9	3.3	2.7–4.0
Depression	1	2.8	2.3–3.3	3.8	3.2–4.5
Burnout	1	3.2	2.7–3.8	3.9	3.3–4.6

^a^Adjusted for age and occupational group

**Table 7:** The effect of three levels of need for recovery (NFR) on scores for single items on health behavior in terms of number of times away from work because of sickness during the past 12 months, number of times using vacation time, flexi-leave, or compensatory leave instead of reporting sick when ill, and number of times going to work despite feeling ill during the last 12 months

Single item (score range)	Low NFR group, mean	SD	In- between NFR group, mean	SD	High NFR group, mean	SD	*df* between groups, and within groups	*F* value	*P* value
Number of times away from work due to sickness (0–10 times = 1–5 points)	**1.6**	**0.6**	1.8	0.8	**2.1**	**0.9**	2, 1256	41.526	0.000^a^
Vacation time or flexi-time leave taken instead of sick leave (n.a., 0 times = 1, and 1–5 times = 2–4 points)	**1.11**	**0.35**	1.22	0.49	**1.36**	**0.7**	2, 1254	20.777	0.000^a^***
Worked despite need for sick leave (0–5 times = 1–5 points)	**1.8**	**1.0**	2.4	1.6	**3.1**	**1.3**	2, 1261	104.736	0.000^a^
Intense exercise per week (0– times)	**1.9**	**1.9**	1.5	1.5	**1.5**	**1.7**	2, 1261	6.795	0.001^b^
Medium- intensity exercise per week (0– times)	**3.0**	**2.9**	2.9	3.2	**2.6**	**2.6**	2, 1182	1.027	0.359 n.s.
Light exercise per week (0– times)	**2.9**	**3.1**	3.2	3.5	**2.9**	**2.7**	2, 1132	1.447	0.236 n.s.
Physical activity with increased heart rate/sweating per week (1–3)	**2.2**	**0.8**	2.1	0.8	**1.9**	**0.8**	2, 1257	8.777	0.000^a^**

Mean scores and standard deviation (SD) are given for all scales and single items. The measurements from the two extreme cluster groups are presented in bold numbers

*na* not applicable

^a^The effect of NFR was significantly different between all three groups, with *p* ≥ 0.000

^a^**The effect of NFR was significantly different between the high and the low NFR group, with *p* ≥ 0.000. Between the low NFR group and the in-between NFR group, the level of significance reached *p* ≥ 0.05. Between the high NFR group and the in-between NFR group, the difference was not significant

^a^***The effect of NFR was significantly different between all three groups, with *p* ≥ 0.05

^b^The effect of NFR was significantly different between the high NFR group and the low NFR group, with *p* ≥ 0.05. The difference between the high NFR group and the in-between NFR group was not significant (n.s.)

## How are NFR cluster groups related to health-related behaviors?

There was a significant effect of NFR group on the items of absenteeism and presenteeism during the last 12 months. Concerning times away from work due to sickness, all three NFR groups gave a range of responses of between zero and three times, with most responses from the high NFR group indicating one to three times. Concerning using vacation time or flexi-leave rather than sick leave when sick, the range was very narrow, with most responses indicating no occurrence, and a minority of cases in the high NFR group reporting one to two occurrences. The item on having worked despite need for sick leave yielded a mean response of two to three times for the high NFR group, while the other groups responded with zero to one times.

Concerning exercise habits, the low NFR group showed a higher score than the other groups concerning physical activity with increased heart rate/sweating per week. The mean scores of all three groups converged around the response option “Sometimes”.

## What is the impact of occupationally characteristic on NFR after work?

Table 8 displays the different occupational characteristic and how work demands, compensatory strategies, and effort-moderating and cumulative load reversing resources for doing the job explained between 40 and 53% of the variance in NFR. In the total group, the explained variance in NFR was 44%. A similarity between the groups was that the demand compensating strategy of thinking of work, while off work concerned all occupations together with the effort-reversing effect on NFR from ROs. Three of the groups represented high rates of occupational injury and were similar in that Quality of work made a strongly reversed contribution to explaining NFR. In the architects and engineers group, the largest explained variance in NFR came from coping with demands in terms of thinking of work when off work, followed by Quantitative demands, and in the reversed direction, the effort modifying resource Influence. Among the home care nurses, thinking of work when off work and coping with demands by lowering the quality of work explained somewhat more of the NFR than in the reversed direction the effort-reversing resources of ROs and effort modifying Quality of work together. Emotional demands contributed to NFR in hospital nurses and in the total group. The predictors Work pace and Emotional demands, together with all of the compensating strategies except for skipping breaks and reversely, Cognitive demands, RO, Support from colleagues, and Quality of work mean contributed to NFR in the total group. In the total group, the NFR reversing scale of Quality of work was the most important predictor. Regarding the occupational characteristics of hospital nurses and also the total group, Support from colleagues made a significant contribution in terms of reducing work-related fatigue.

**Table 8:** Models of four professional groups; stepwise linear multiple regression with need for recovery as dependent variable and quantitative demands, work pace, cognitive demands, and emotional demands together with strategies of working more intensively to finish, skipping breaks to finish, lowering the quality of work, thinking of work when off work, taking work home to complete, influence, recovery opportunities, support from colleagues, support from supervisors, Quality of work, and age as predictor variables. Occupational characteristics of architects/engineers, carpenters, hospital nurses, and home care nurses, and a total group that included also other occupational characteristics

Variables	Architects/civil engineers *N* = 218		Carpenters *N* = 150		Hospital nurses *N* = 520		Home care nurses *N* = 103		Total group *N* = 1215	
	Beta	*P* value	Beta	*P* value	Beta	*P* value	Beta	*P* value	Beta	*P* value
Work demands										
Quantitative demands	0.240	0.001								
Work pace	0.158	0.021			0.120	0.002			0.114	0.000
Cognitive demands									− .071	0.007
Emotional demands					0.082	0.033			0.101	0.000
Effort-moderating resources										
Influence	− .163	0.005								
Support from colleagues					− .100	0.005			− .063	0.006
Support from supervisor										
Quality of work			− .365	0.000	− .265	0.000	− .203	0.031	− .255	0.000
Cumulative effort-reversing resource										
Recovery opportunities	− .138	0.030	− .261	0.000	− .144	0.000	− .223	0.005	− .235	0.000
Compensating strategies										
…work more intensively in order to finish			0.274	0.000					0.067	0.008
…skip breaks or lunch to finish										
…lower the quality of my work in order to finish					0.103	0.009	0.276	0.003	0.072	0.004
…think of my work even when I am off work	0.258	0.000	0.186	0.004	0.251	0.000	0.301	0.000	0.219	0.000
*…*take work home and complete in my spare time					− .097	0.011				
Added in step 2										
Age	− .058	0.568	− .043	0.604	0.38	0.394	− .043	0.679	− .043	0.134
Model summary										
Total adjusted R	0.420		0.532		0.396		0.476		0.437	
Change from step 1 to step 2										
F of regression equation	32 159		43 339		430 441		23 751		94 636	
Significance of F		0.000		0.000		0.000		0.000		0.000

## Discussion

The comparison between the two groups that either often experienced NFR (high NFR) or sometimes experienced NFR(in-between group) and the low NFR group showed that the two former groups at two different levels reported distinctly higher demands and distinctly lower available effort-moderating and cumulative effort-reversing resources at work than the low NFR. The group less than sometimes experiencing NFR (low NFR) group presented an inverse pattern of lower demands and higher effort-moderating and cumulative effort-reversing resources. Moreover, the high NFR group and the in-between group at two different levels both contrasted to the low NFR group that more rarely used work-demand compensatory strategies such as skipping breaks to finish what needs doing at work. Concerning health effects, a salutary meaning of a low NFR could be validated, while this group negated regular health effects. In contrast, a high NFR and an in-between level of NFR value at two different levels meant experiencing symptoms on a regular basis and an unhealthy pattern of up to a fourfold magnitude concerning health effects compared to the low NFR group. During the last 12 months, the health behaviors concerning a real need for sick leave to some degree differed between the cluster groups, while the contrasting high NFR group had worked despite this need two to three times as compared to zero to one time in the other groups. A minor contrast between the groups concerned exercise habits where the low NFR group was more often physically active at an intensive level.

The fifth research question concerned the impact on NFR from occupational characteristic of four narrowly specified occupational groups. In the same order of magnitude and in size, the most important impact on NFR came in the group of engineers and architects from Thinking of work when off work and Quantitative demands. Concerning the carpenters, this impact stemmed from Lack of opportunity making a good quality job, working more intensively to finish and Lack of recovery opportunities. In the group of hospital nurses, this impact comprised of Lack of opportunity making a good quality job and Thinking of work when off work and among Home care nurses from Thinking of work when off work, Lowering the quality of the job to finish, Lack of recovery opportunities, and Lack of opportunity making a good quality job. In the non-academic groups, the total of occupational characteristics explained 48–53% of the variance in NFR. The corresponding figure in the higher educated groups was 40–43%.

The suggestion of Fig. 1 concerning a quantity of NFR corresponding to a weight of effort that in turn relied on the balance between (moderating and effort revering) resources and work demands (added by effort from compensating strategies) was largely confirmed. The different situations of the three levels of NFR cluster groups support this dynamic also concerning additional effort during work from residual fatigue which in the model is suggested.

NFR equals accumulation of effort and fatigue. If not nugatory, this gradually also creates a situation where fatigue compensating effort results in a successively more vulnerable states of overdraft fatigue during the day at work, NFR. In addition to being a vulnerable state of mental fatigue, compensatory effort may alter the functioning of the brain in the direction of the effort being both very costly and inefficient (e.g., Durning et al. 2013; Tanaka et al. 2015). This costly adaptation of the fatigued brain alone constitutes strong arguments for internal (“at work”) successive recovery.

Five items aimed at *how* the work demands were managed in terms of, for example, working faster to finish the tasks. Four items discriminated between the two levels of salutary and unhealthy NFR, which supports the model of Fig. 1. Concerning these compensating strategies, the regression analysis showed that thinking of work when off work made the most prominent contribution to NFR from all recorded sources of demands at work. Is this then a strategy managing work demands or is this rather a load reaction meaning a symptom of fatigue? In the current result, thinking of work when off work relates to fatigue (NFR), which is not the case with unemotional problem-solving pondering about work after work (Kinnunen et al. 2017). This suggests that in the current result thinking of work while off work refers to emotional rumination, by which is meant involuntary unwanted thought about work, including emotional reactions. Emotional rumination implies also a state of weakened self-controlling cognitive resources (Cropley et al. 2016). The results on the load reaction of thinking of work when off work support the dynamics of the model in Fig. 1, while emotional rumination is known to tax the cognitive resources needed by the employee doing the job the next day (Cropley et al. 2016), meaning effort from residual cognitive fatigue at work. Emotional rumination is also a health risk with a known physiological underpinning (Cropley et al. 2017) including explanatory power concerning cardiovascular disease. This latter course refers to a latency time process and is not covered by the current model.

Research question number two and four concerned some effortful behavioral measures that corresponded to three levels of work demands and NFR. Those who were more fatigued also more often used work-demand compensating strategies and vacation or flex time instead of sick leave and they more often went to work when sick. Thereby, fatigue appears to entail problem-solving that creates more effort both inside and outside the work place. In parallel, researchers have identified a natural inclination to protect and conserve necessary higher cognitive resources (e.g., problem-solving) by not depleting them (Muraven et al. 2006). Then means being regularly fatigued that this protective inclination is set aside? In this direction, speaks that also thinking of work when off work relates both to fatigue (Kinnunen et al. 2017) and failing cognitive controlling resources (Cropley et al. 2016). These tentative more global effects from fatigue on cognition create strong arguments for a balanced and healthy work situation where the employees could maintain their personal resources throughout the day.

### Concurrent effects on health means validation of a salutary NFR

The latency of different mental load reactions from stress is very short, as described from experimental stress research (e.g., Vinkers et al. 2013; Soares et al. 2012), and the functional fatigue scale NFR mirrors a mean level over 3–4 weeks. The salutary meaning of a low NFR could be validated, while this group negated regular health effects. Also, other studies show that more rarely experiencing NFR comprise less health effects (Schuring et al. 2004). This in turn suggests a possibility of setting a limit value concerning sufficient recovery from work. The current data gathering is also a fundament in a newly developed web instrument that records NFR together with conditions of work (see, e.g., Wentz et al. 2018a, b). The instrument could be used in a combined monitoring of work and load reactions. In the current result, the salutary NFR cluster is demarcated by the scores 0–9/33 on the NFR scale. These same limit values have through linear regression procedures been identified as not harmful load reactions after work (Wentz et al. 2018a, b).

Regarding a potential direct or mediated effect on health from NFR, De Lange et al. (2009) found that a high level of work demands meant both a gradual increase in diurnal fatigue and a gradual failing function of sleep. In contrast to (increased) diurnal fatigue, some serious consequences for both physiological balance and mental health from a failing function of sleep are already known (Akerstedt 2006). Similar consequences may apply to unfavorable states of fatigue, but seem to be less investigated. However, diurnal fatigue is more predictive of sick listing than impaired sleep. In tandem, impaired sleep was suggested as among the causal factors behind the prediction from fatigue (Akerstedt et al. 2007). A more direct link between fatigue and health effects such as impaired sleep or undermining of physiological balance and mental health could be a compulsory increase in effort at work based on being fatigued. Links such as these need further investigation.

### From general patterns of occupational characteristics regression profiles and interventions

In the total study group, having the opportunity to make a good Quality of work in the reversed direction made the largest contribution to NFR. Also, among carpenters and hospital nurses, lack of the moderating resource Quality of work had the largest impact on NFR. Also, in home care nurses, lack of Quality of work opportunities was very important in explaining NFR. To cope with the total amount of tasks, employees may have had to compensate by lowering the quality of work. This conflicting situation is mirrored by Pousette (2001) who underlines that in caring professions, lack of resources for doing the job as in “do good for others” results in stress.

This effort-moderating resource of making a good quality of work logically concerns the resource dimension of creating meaning from work. This is an important finding with progenitors also from other research. From Swedish conditions, Aronsson and Lindh (2004) reported that sufficient resources doing the job and experience of a good quality level predicted the so-called long-term health, as in low sickness absenteeism and low sickness presenteeism. Concerning the effort-reversing effects on NFR of the resource ROs, there was an emphasis on groups managing both mental and physical load, as in carpenters and home care nurses, which confirms earlier findings on a higher need for recovery from both mental and physical load (Sluiter et al. 2000).

Interpretable profiles were that among the engineers/architects cognitive fatigue in terms of reduced attentional control meant thinking of work when off work, which made the biggest contribution to NFR. This particular load reaction was validated by Quantitative demands and Work pace contributing in the same direction to NFR. In this situation, the work process moderating or reversing scales of influence and ROs also played a part. In carpenters, four major predictors explained NFR, where Quality of work may moderate and ROs in part reverse effort from the strategy of working fast to finish together with thinking of work when off work. In this group, NFR was explained to the highest degree (53%). Regardless of the differences in the level of education, there are important similarities between the caring professions. These similarities include often working part time. The patterns of effort starting from the compensating strategy of lowering the quality of work was a very pronounced source of effort in home care nurses and concerned hospital nurses, as well, wherein both professions’ effort could be moderated by Quality of work. Thinking of work when not at work was an important source of effort in both caring professions. These tripod mechanisms may rely on the conflict in caring professions described by Pousette (2001) above. Among hospital nurses, work pace predicted NFR. In home care nurses, RO could play an important part in reversing NFR. In hospital nurses and total group, Support from colleagues made a significant contribution in terms of potentially reducing work-related fatigue.

Interventions in the engineer/architect group need to aim at sufficient resources in terms of staffing for doing the job to discontinue quantitative overload forcing a work style marked by intensity and absorption (Allen et al. 2002) during work in turn promoting ruminating thoughts about work after work. Sufficient staffing may also increase influence over the work process. In addition, concerning carpenters, sufficient staffing for doing the job may cause the compensating strategies to end and create a situation of feeling pride in work in a natural way. In the caring professions, again, sufficient staffing may adjust a situation where lack of opportunities for doing a good job severely challenges the effort-moderating and effort-reversing resources in the work place. In tandem, sufficient staffing could prevent a fatiguing process that gives rise to thinking of work when off work.

Regarding resources for doing the job, the lack of the effort-reversing effect of ROs on NFR to a larger degree concerned groups managing both mental and physical load, namely carpenters and home care nurses. In the occupational groups with higher rates of occupational injury, lack of opportunity to perform a good Quality of work was very important in explaining NFR.

## Limitations

The present result concerns taking a helicopter perspective on the diverse exposures from psychosocial working conditions in diverse professional settings in four narrowly defined occupational groups. This meant that the exposures were translated into the occurrence of a load reaction of functional fatigue at the end of the working day, in terms of NFR. Three levels of NFR groups was produced through cluster analysis and labeled low, high, and in-between NFR. Thereafter, the situation of each group concerning the occurrence of NFR, work demands and resources, compensatory strategies, health, and health behavior was depicted by level descriptors from the different scales’ response options.

This baseline study is cross-sectional in nature and included professions that are largely female or male-dominated, with one profession (engineering) representing a low rate of occupational injury. We had a low response rate and more women than men participated in the study, and conclusions from this study may have to be drawn with these limitations in mind. At the same time, men and women are found to be equally sensitive to strain damage or psychological problems from physical or mental strain. S.c gender differences could instead be explained from various gender typical occupational characteristics (Swedish Research Council for Health, Working Life and Welfare (2016). Accordingly, our calculations concerned the associations between work and health of the individuals. However, the participants cannot be regarded as representing the occupational group; rather, our study population should be viewed as having four diverse occupational characteristics.

In conclusion, high, in-between, and low NFR related to effort from three corresponding distinctive levels of work demands, stress responses of high work-demand compensatory strategies and unfavorable health behaviors such as repeatedly working while ill. Three distinctive levels of resources at work that could moderate effort or reverse cumulative effort during work were in inverse proportion related to high, in-between and low NFR. In the current result, the defined low level of NFR meant also to negate regular functional effects on health. This finding could guide the setting of limit values regarding a salutary level of NFR. In the total study group, the most important predictors of NFR were the effort moderation from being able to make a good Quality of work, effort reversal from RO, and additional effort from thinking of work when off work. Concerning the occupational groups, meaningful patterns of predictors appeared that could also guide interventions. An implication for future research on work and health could be to broaden the perspective on the spectrum of workload and resources to do the work. This could also have an educational significance in interventions.
